# Morphology of Mitochondria in Syncytial Annelid Female Germ-Line Cyst Visualized by Serial Block-Face SEM

**DOI:** 10.1155/2020/7483467

**Published:** 2020-01-07

**Authors:** Anna Z. Urbisz, Sebastian Student, Małgorzata A. Śliwińska, Karol Małota

**Affiliations:** ^1^University of Silesia in Katowice, Institute of Biology, Biotechnology and Environmental Protection, 9 Bankowa Street, 40-007 Katowice, Poland; ^2^Institute of Automatic Control, Silesian University of Technology, 16 Akademicka Street, 44-100 Gliwice, Poland; ^3^Nencki Institute of Experimental Biology of Polish Academy of Sciences, Laboratory of Imaging Tissue Structure and Function, 3 Pasteur Street, 02-093 Warsaw, Poland

## Abstract

Mitochondria change their morphology and distribution depending on the metabolism and functional state of a cell. Here, we analyzed the mitochondria and selected structures in female germ-line cysts in a representative of clitellate annelids – the white worm *Enchytraeus albidus* in which each germ cell has one cytoplasmic bridge that connects it to a common cytoplasmic mass. Using serial block-face scanning electron microscopy (SBEM), we prepared three-dimensional ultrastructural reconstructions of the entire selected compartments of a cyst at the advanced stage of oogenesis, i.e. the nurse cell, cytophore, and cytoplasmic bridges of all 16 cells (15 nurse cells and oocyte). We revealed extensive mitochondrial networks in the nurse cells, cytophore and mitochondria that pass through the cytoplasmic bridges, which indicates that a mitochondrial network can extend throughout the entire cyst. The dynamic hyperfusion state was suggested for such mitochondrial aggregations. We measured the mitochondria distribution and revealed their polarized distribution in the nurse cells and more abundant accumulation within the cytophore compared to the nurse cell. A close association of mitochondrial networks with dispersed nuage material, which seems to be the structural equivalent of a Balbiani body, not described in clitellate annelids so far, was also revealed.

## 1. Introduction

Mitochondria are highly dynamic organelles that are primarily specialized in producing energy by generating most of the adenosine triphosphates (ATP). To maintain cells in proper energetic metabolism and homeostasis, they also play other important roles in intermediary metabolism, calcium signaling and apoptosis [1–5]. In this context, the morphology of mitochondria, their spatial distribution and activity are changeable in cells depending on their temporary requirements. The dynamism of mitochondria is the result of two opposing processes: fusion, which leads to their merging into larger mitochondrial networks and fission, which is the process of separation of an individual mitochondrion from the network. The morphology of mitochondria depends on the balance between these processes [3, 6–16].

One fundamental role that has been suggested for mitochondrial networks is that they enable communication between organelles in order to facilitate access to or exchange of the products of mtDNA expression [3, 12, 17]. Fusion can also buffer the transient defects that arise in individual mitochondria. Mitochondrial fission, on the other hand, can serve as a mechanism of mitochondrial selection, which causes the elimination of mitochondria with deleterious components sorted inside them [2, 3, 18]. Mitochondrial networks have been found in different types of cells. Their genetic basis and the molecular mechanisms of how they fuse into larger systems or split into single organelles have been intensively studied in model organisms such as yeast and mammalian tissue cell cultures [18–22].

Analysis of mitochondrial dynamism and the distribution of mitochondrial networks in different cell systems have mainly been based on confocal fluorescent microscopy [21, 23–27] or high resolution 4Pi microscopy [28, 29]. Studies to reveal the complexity of the morphology and distribution of the mitochondria at the ultrastructural level that are not based on single ultrathin sections but in the form of three-dimensional (3D) reconstructions are rare [30]. Recently, the mitochondrial network has been shown at the ultrastructural level in the Balbiani body (Bb) in the growing oocytes of an insect, *Thermobia domestica *[31]. Bb (mitochondrial cloud), which is a characteristic organelle complex that occurs in many invertebrate and vertebrate species [32], is suggested to be involved in the selective elimination of dysfunctional mitochondria during oogenesis [31]. However, the oogenesis of *Thermobia* represents only one example among many in which future egg cells develop individually, while in many animals, formation of the egg cells takes place in a groups of connected germ cells (see below).

Regardless of the technique used, the studies devoted to mitochondrial distribution, dynamism and energetic processes were based on a number of individual somatic cells, such as neurons, pancreatic *β*-cells, and hepatocytes [28, 33, 34]. Even if we take into account the syncytial systems, in which these mitochondrial issues were studied (e.g. rat cardiomyocytes, rat and human skeletal muscles, fruit fly blastoderm embryo) [34–38], there are still syncytial systems of somatic cell lines.

As mentioned above, in many animals, temporary groups of interconnected germ cells, which are in fact functional syncytia, occur during oogenesis and spermatogenesis [33–36]. These systems are known as germ-line cysts, nests or clones and are highly variable in their spatial arrangement (of which the best known are the highly branched cysts in fruit fly ovaries) [30, 37–41]. Additionally, in the case of oogenesis, the germ cells clustered into cysts may not have the same developmental potential. As oogenesis progresses, some of the interconnected germ cells (called nurse cells; NCs) begin to work for the other cells (oocytes, i.e. future egg cells) by delivering different classes of macromolecules (mainly RNAs) and organelles, including mitochondria, to the growing oocytes [40–43]. The exchange of organelles and macromolecules (cytoplasm sharing) between interconnected cells is enabled by the presence of the broad (average of 10 *μ*m in *D. melanogaster*) cell junctions that are called intercellular bridges or ring canals (RCs) [42, 44–46]. Although NCs do not have the potential to develop into egg cells, usually they are highly specialized, polyploid cells, and after they have fulfilled their functions, they are eliminated from the cysts via programmed cell death – apoptosis [34, 37, 40, 42–50].

The germ-line cysts are an interesting model for the study of syncytial systems formation and functioning, including the organization and dynamism of the mitochondria. Do the mitochondria form the networks within a single clustered cell or are the mitochondria individual organelles? If networks exist, do they extend between cells via the RCs and can the mitochondria make contact with the mitochondria in the other compartments of a cyst, or they are rather isolated? The present paper is the first attempt to analyze the mitochondrial dynamism, morphology, and distribution in such syncytial compartments. Additionally, we have selected the unique syncytium in which as a rule, the germ cells are not connected directly to each other, as in cysts in the model organisms *D. melanogaster* and *Xenopus laevis* [30, 39, 51], but instead of this each germ cell has one RC that connects it to a common and anuclear mass of cytoplasm that is located in the center of the cyst (the cytophore) [52–54]. Such cysts equipped with a central cytoplasmic mass (of different morphology) have been found in clitellate annelids [52, 53], echiuran annelids [55, 56], *Caenorhabditis elegans* and other nematodes [57–61], and in oribatid mites [62, 63]. Moreover, in the cysts we have chosen, the germ cells are differentiated in their developmental fate and two cell categories – the 15 NCs and the one growing oocyte – are gathered together. Such a structural type of female germ-line cysts is represented in the white worm *Enchytraeus albidus* (belonging to clitellate annelids) and this was chosen for our research purposes.

To visualize the morphology and distribution of the mitochondria within a germ-line cyst, we prepared three-dimensional ultrastructural reconstructions using the SBEM technique. This technique is based on installation of the ultramicrotome inside the SEM chamber, which enables sequential ultrastructural micrographs of analyzed structures to be obtained with EM resolution, which is the basis for the 3D visualizations [64, 65]. It is also worth mentioning that we did not concentrate solely on a fragment of a cell (as in the Bb that has been reconstructed in *T. domestica *oocytes) [31] or even at a single cell (as in several other studies) [64, 66], but we reconstructed the entire selected compartments of a germ-line cyst, i.e. the NC, cytophore and RCs that interconnect all 16 germ cells. Among the germ cells, we concentrated on the NCs and have not yet reconstructed the distribution of the mitochondria within a growing oocyte due to the fact that it is a very extensive cell (average size ~200 *µ*m) [52] and should be analyzed separately. It is also worth mentioning that in *E. albidus*, the NCs are not as highly specialized as in the other species (e.g. *D. melanogaster*) because of the absence of polyploidization (only 4 C levels of ploidy) and the absence of the apoptotic process as they are engulfed by the oocyte at the end of oogenesis. These cells are connected to the common cytophore by the broad channel of the ring canal (~4 *µ*m in diameter) [52]. Similar to the NC in other species, the NC in the germ-line cysts in *E. albidus* are also relevant in the development of the oocyte [52].

In our study, SBEM was suitable to study the morphology and distribution of these organelles in detail and together with the classical light and transmission electron microscopy showed the polarization of NCs, which was manifested by the extensive mitochondrial networks that occur near the nucleus in the pole of the cell opposite to the RC and a dense aggregation of mitochondria in the cytophore. In the cytophore and within the RCs, the mitochondria can also fuse, which indicates that the mitochondrial networks seem to extend throughout the entire germ-line cyst. Our observations suggest that the mitochondria in the selected NC are in a dynamic hyperfusion state. The cyst can thus exchange these organelles between particular compartments, which indicates the presence of the functional mitochondrial syncytium. Additionally, we also revealed the close association of the NC mitochondrial aggregations with the dispersed nuage material (specific for the germ cells) as well as an abundance of other cell organelles (Golgi complexes, endoplasmic reticulum) that seems to be the structural equivalent of the Bb, the organelle complex important in proper formation of the oocyte and the future embryo [67, 68], which is found in other animals, and which has not yet been described in *E. albidus* and other annelids.

## 2. Results

### 2.1. Germ-Line Cyst Architecture

**Figure 1:**
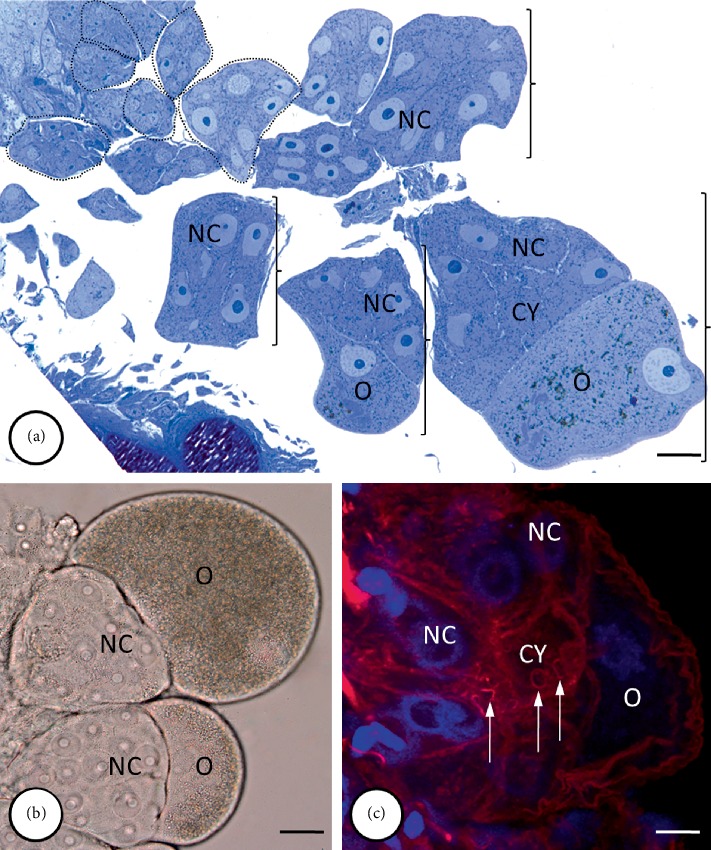
Organization of the germ-line cysts in the *E. albidus* ovary. (a) Several almost spherical cysts are visible within the entire ovary. The cysts are composed of germ cells in successive stages of oogenesis. Initially, all of the germ cells within the cyst were undifferentiated (dotted line), but in the later stages (*bracket*), one growing oocyte (*O*) and several of the 15 smaller nurse cells (*NC*) were visible in the cysts; *Cy* – cytophore. (a)–(c) The organization of the germ-line cysts was always the same: the germ cells were located at the cyst periphery (both the nurse cells – *NC* and the oocyte – *O*) and each one was connected to the centrally located mass of cytoplasm, the cytophore (*Cy*) via one open cytoplasmic channel called a ring canal (*arrows*). Rhodamine-conjugated phalloidin staining showed that the ring canal rim was rich in F-actin. Light microscopy, (a) Epon semi-thin sections stained with methylene blue, bar = 10 *µ*m; (b) whole-mounted preparation, Nomarski interference contrast, bar = 30 *µ*m; (c) the cysts double stained with DAPI (blue) and rhodamine-conjugated phalloidin (red), bar = 5 *µ*m.

**Figure 2:**
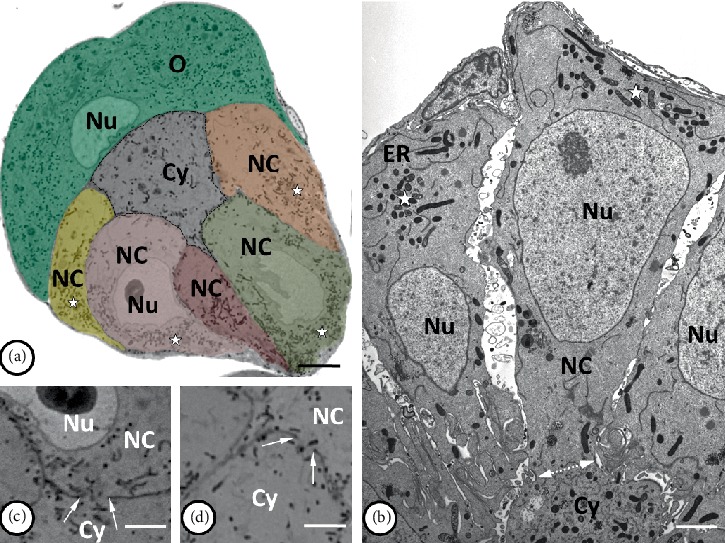
Organization of the mitochondria in syncytial germ-line cysts. (a) and (b) The accumulations of mitochondria (*stars*) are located mainly in the one pole of the nurse cells (NC) opposite the ring canal (*bilateral dotted arrow* in (b)), while in the cytophore (*Cy*) the mitochondria were evenly scattered in the cytoplasm. Nuclei of the nurse cells and oocyte (*O*) are marked as *Nu*; tubules of the endoplasmic reticulum as *ER*. (c) and (d) the mitochondria (stained black) are visible passing through the ring canals (*arrows*) connecting the nurse cells (*NC*) to the cytophore (*Cy*). *Nu* – nurse cell nucleus. (a, c, d) Light microscopy, Epon semi-thin sections stained with methylene blue. (a) Bar = 73 *µ*m; (c, d) bar = 30 *µ*m. (b) Transmission electron microscopy, Epon ultrathin section; bar = 2 *µ*m.

The architecture of the *E. albidus* female germ-line cysts was described recently [52]. The individual cysts within the ovary represent the successive stages of oogenesis. Initially, all of the interconnected cells are morphologically identical (until the end of pachytene/early diplotene of the 1^st^ meiotic prophase) (Figure 1(a)) but later, the germ cells differentiate depending on their fate and develop into two cell types: 15 nurse cells (NCs) and one oocyte (Figures 1, 2(a), and 4(a)). Only the oocyte continues meiosis, grows considerably and becomes the egg cell, while the NCs function as supporting cells and deliver at least cell organelles to the oocyte [52, 69]. The architecture of the cysts is always the same – the germ cells are located at the cyst periphery and they are connected to a common mass of cytoplasm (cytophore) that is located in the center of the cyst. The cytophore is a small, roughly spherical cytoplasmic mass that is devoid of nuclei and each germ cell is connected to it by one ring canal (RC) (Figures 1(a), 1(c), 2(a), and 4(a)). The RCs are open cytoplasmic channels that are about 4 *µ*m in diameter (in the case of the NCs) and are stabilized by a ring-like inner rim, which is rich in F-actin (Figure 1(c)). The structure and ultrastructure of the cysts have been described in detail [52, 69, 70]. Here, we analyzed the cysts in which the oocyte had already started to absorb the yolk (the so-called early vitellogenic oocyte) and is clearly distinguishable from the remaining 15 cells, which do not absorb the yolk and serve as supporting cells (Figures 1, 2(a), and 4(a)). In 3D we reconstructed the mitochondria and other selected structures (e.g. nuage material) at the ultrastructural level in one randomly selected NC, the entire cytophore and all 16 of the RCs.

### 2.2. Arrangement of Mitochondria

**Figure 3:**
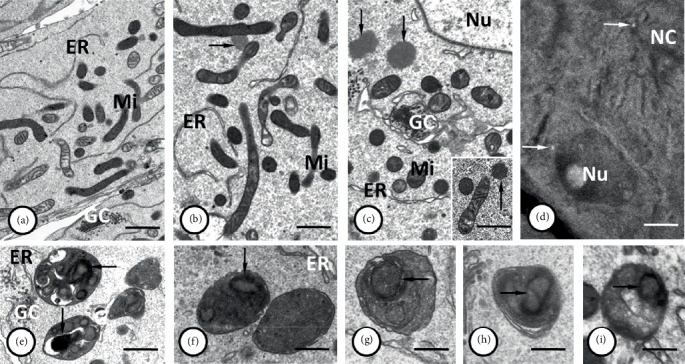
(a–c) Accumulations of mitochondria (*Mi*) together with the tubules of the endoplasmic reticulum (*ER*) that enclosed the Golgi complexes (*GC*) are visible in the nurse cell pole opposite the ring canal, near the cell nucleus (*Nu*). Note the two distinguishable kinds of mitochondria with electron-dense and electron-lucent matrix, both of them possessing numerous cristae. Granulo-fibrillar nuage material (*arrows*) was observed close to mitochondrial aggregation within both nurse cells and cytophore (inset). (d) Thioflavin T staining showed that the nuage material was rich in amyloid-like proteins (*arrows*). *NC* – nurse cells; *Nu* – nurse cell nucleus. (e–i) Autophagosomes in nurse cells containing the remnants of cell organelles including the mitochondria (*arrows*). Tubules of endoplasmic reticulum – *ER*; Golgi complexes – *GC*. (a–c), (e–i) Transmission electron microscopy, Epon ultrathin sections; (a) bar = 1.4 *µ*m; (b), (c), (e) bar = 0.9 *µ*m; (f) bar = 0.5 *µ*m; (g, h) bar = 0.4 *µ*m; (i) bar = 0.3 *µ*m. (d) Steedman wax sections stained with thioflavin-T; bar = 45 *µ*m.

**Figure 4:**
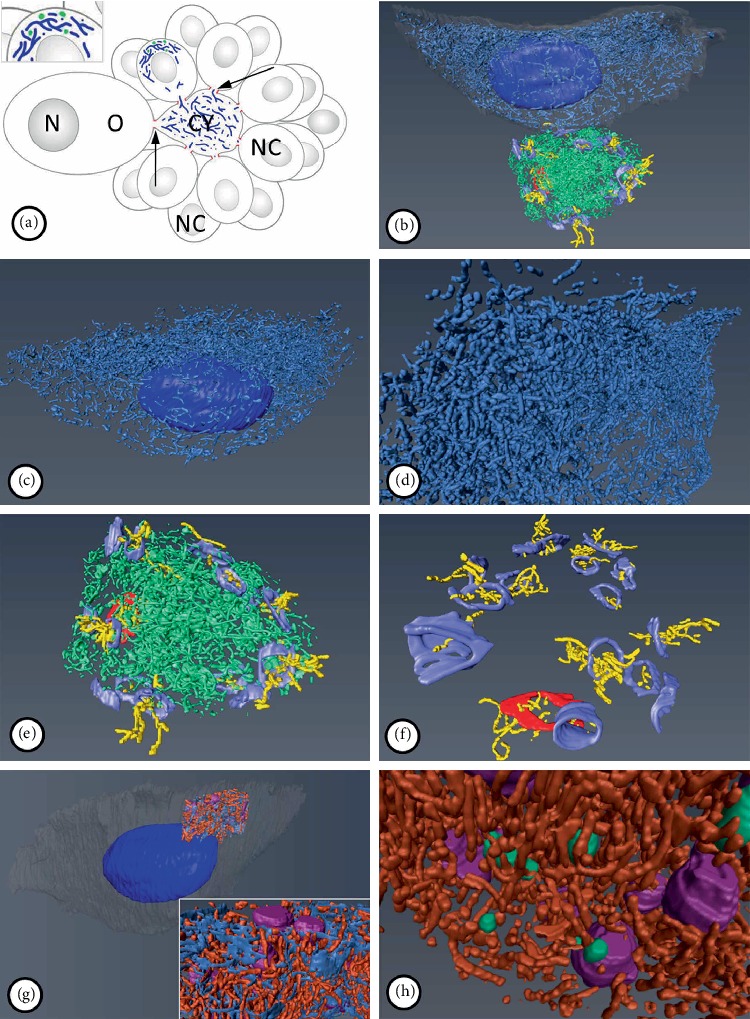
(a) Diagram showing the general organization of a germ-line cyst with a reconstructed nurse cell (*NC*), cytophore (*Cy*) and ring canals (*arrows*). Mitochondria are shown in blue, nuage in nurse cell as green dots (inset). (b–h) Three-dimensional reconstructions of the selected components in the analyzed germ-line cyst: general view with reconstructed mitochondria in the nurse cell and cytophore (b), nurse cell mitochondria (c, d), cytophore mitochondria with the 16 ring canals (e), ring canals with the mitochondria passing through them (f), legend: *gray* – nurse cell outline, *dark blue* – nurse cell mitochondria, *light green* – cytophore mitochondria, *blue* – nurse cell nucleus, *light blue* – ring canal rims of nurse cells, *red* – ring canal rim of oocyte, *yellow* – mitochondria passing through the ring canals. Reconstruction of the fragment of the nurse cell with the nuage (g, h), legend: *light green* – nuage material, *purple* – Golgi complexes, *dark red* – mitochondria of the nuage area, *light blue* – endoplasmic reticulum.

**Figure 5:**
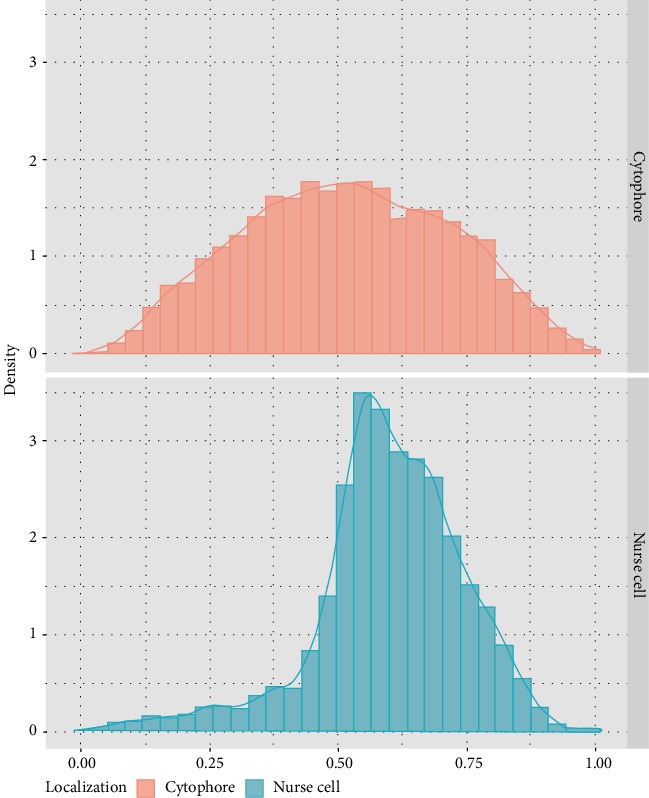
Diagram of distribution of mitochondria in the nurse cell and cytophore that were selected and reconstructed in 3D, in relation to the ring canal.

**Figure 6:**
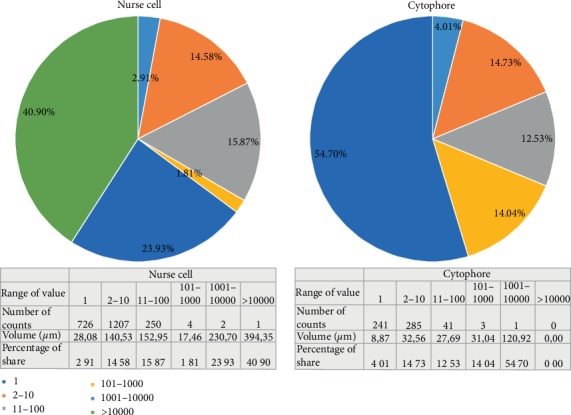
Diagram of level of connectivity of mitochondria in the nurse cell and cytophore. The diagram shows the percentage of groups of mitochondria based on their level of connectivity. Individual ranges of values mean the number of single mitochondria included in the groups, where 1 means the single (individual) mitochondrium. The range of values were estimated based on the measured volume of single mitochondrium which was 0.0259 ± 0.01 *µ*m^3^ (*n* = 25).

An analysis of the images using a light and transmission electron microscope (TEM) as well as the data obtained by ultrathin cutting and serial scanning of the block surface (SBEM) revealed numerous mitochondria in the NC and the cytophore (Figures 2, 3(a)–3(c), and 4, Supplementary Video 1–5 online). The measurement of the total mitochondria volume showed 964 *µ*m^3^ in the selected NC and 221 *µ*m^3^ in the cytophore. When the measured volumes of mitochondria per volume of NC' cytoplasm and cytophore were compared, it was clearly visible that there was 25% more abundant accumulation of the mitochondria in the cytophore than in the NC (Table 1). In the NCs, mitochondria were distributed unequally in the cytoplasm and more abundant accumulations were located around the nuclei and especially on the cell pole opposite to the RCs (Figures 2(a), 2(b), and 4(b)–4(d), Supplementary Videos 1 and 2 online). Fewer mitochondria (see in Figure 5) were near the RCs; thus there was an evident gradient of the distribution of the mitochondria in the axis RC – the cell pole opposite to the RC (Figures 2(a), 2(b), 4(a)–4(d), and 5) – and thus the NCs were clearly polarized. In the NC that was selected for reconstruction, the aggregations of mitochondria were linked together and formed extensive networks that consisted of long and branched mitochondria, which were clearly visible in the 3D reconstructions and were also observed in the ultrathin sections in the reconstructed cell and other NCs in the cysts (Figures 2, 3(a)–3(c), and 4(b)–4(d), Supplementary Videos 1 and 2 online). It should be noted that among the interconnected mitochondria, small, individual and bean-shaped mitochondria were also revealed (Figures 4(b)–4(d), Supplementary Videos 1 and 2 online). We have measured the volume of single (individual) mitochondrium and the mean value was 0.0259 ± 0.01 *µ*m^3^ (*n* = 25). The number of these individual mitochondria in the NC was 726 and it stated about 3% of the total NC mitochondria volume. To check the level of the connectivity of the mitochondria, we have measured how many of such an individual mitochondria are included in the longer interconnected ones. The analysis have shown 3 large (fused) mitochondria (consisted of over 1.000 individual mitochondria) which were about 65% of the total NC mitochondria volume, and numerous also connected but smaller ones (stated about 32% of the total NC mitochondria volume) (Figure 6).

As mentioned above, there was more abundant accumulation of mitochondria within the cytophore than in the analyzed NC (Table 1). An analysis of localization of the mitochondria showed that they were distributed evenly within the cytophore cytoplasm (Figures 2(b), 4(b), 4(e), and 5, Supplementary Videos 1–3). A 3D analysis of the cytophore also revealed that the mitochondria were fused and formed a network of long, branched organelles (Figures 4(b) and 4(e), Supplementary Videos 2 and 3 online). Similar to the reconstructed NC, some individual small mitochondria were documented between them (Figures 4(b) and 4(e), Supplementary Videos 2 and 3 online). The measurement of the level of the mitochondrial connectivity has shown one large connected mitochondrium (included 4650 individual mitochondria) which was 55% of the total cytophore mitochondria volume, and numerous smaller connected mitochondria which were 41%, while there were also 241 small individual mitochondria, which was 4% of the total cytophore mitochondria volume. The statement of the measured values and a schematic diagram of mitochondria distribution in the NC and the cytophore are presented in Table 1, Figures 5 and 6.

Mitochondria were also observed in close proximity to the RCs (from the side of the NCs, oocytes and cytophores) as well as within the cytoplasmic canals of the RCs (Figures 2(b)–2(d), 4(b), 4(e), and 4(f), Supplementary Video 2–4 online). 3D reconstructions of each of the 16 RCs showed that the mitochondria within different cyst compartments were in close contact with each other and passed through the RCs cytoplasm, but in two reconstructed RCs there were no mitochondria passing (Figures 4(b), 4(e), and 4(f), Supplementary Video 2–4 online).

During TEM analysis we observed single autophagy vesicles (autophagosomes) within the cytoplasm of NCs and also within germ cells at the earlier stages of oogenesis (cystocytes undiffrentiated yet into nurse cells and oocyte). Autophagosomes contained remnants of organelles, such as mitochondria and Golgi complexes (Figures 3(e)–3(i)).

It should be noted here that during our study we have made multiple attempts to monitor mitochondrial functions and their role in the germ-line syncytium, using available assays. We have tried to use the following: (1) the fluorescent dye JC-1 (5,50,6,60-tetrachloro- 1,10,3,30-tetraethylbenzimidazolcarbocyanine iodide), which is a marker of mitochondrial activity and allows one to detect changes in the electrochemical potential of the inner mitochondrial membrane, (2) active MnSOD antibody (Rabbit Anti-MnSOD Polyclonal Antibody) to detect mitochondrial superoxide dismutase (MnSOD), (3) MitoTracker Orange CMTMRos for mitochondria visualization (on the basis of mitochondrial membrane potential) in live cells, (4) DiOC6(3) to dye the mitochondria and other internal membranes, such as the endoplasmic reticulum of live cells. Despite many attempts and assay modifications all of the dyes used were consistently unable to penetrate the germ cells and cytophore, no mitochondrial staining in their cytoplasm was found. The only cells in which mitochondria were labeled to some extent were growing oocytes. This was probably due to the low permeability of the outer somatic envelope, made up of flattened somatic cells that tightly cover the cyst surface. As was shown in a previous study [52], when the oocyte grows, the somatic envelope that covers it breaks down and probably for this reason the only signal obtained came from the oocytes and somatic cells surrounding the germ-line cysts (Supplementary Figure 1 online). Interestingly, in similar studies, but on male germ-line cysts, labeling in germ cells was achieved [71], but there are no somatic cells that would surround clusters, so reagents could freely penetrate inside the cysts. To compensate for the lack of analysis in fluorescence microscopy we have analyzed the ultrastructure of the mitochondria by transmission electron microscope, using the material processed in the same way as for SBEM, which allows good visualization of the biological membranes. Thus we observed numerous cristae in the mitochondria, although they differ in the electron density of the mitochondrial matrix: one kind of mitochondria possessed electron light matrix, while the other kind of matrix had higher electron density (in these mitochondria the cristae were sometimes hardly visible, due to the similar contrast between the matrix and cristae) (Figures 3(a)–3(c)).

### 2.3. Nuage Material

We also analyzed selected NC components that are closely associated with the mitochondrial network such as the endoplasmic reticulum, Golgi complexes and electron-dense accumulations of unbounded granulo-fibrillar material that are characteristic for the germ-line cells of numerous animals that are collectively called “nuage material” [32, 51, 53, 54]. The use of TEM and analysis of 3D reconstructions obtained by SBEM revealed the occurrence of patches of electron-dense granulo-fibrillar nuage material, scattered among the mitochondria and also contacted directly with them (Figures 3(b) and 3(c), Supplementary Video 5 online). It is worth mentioning that germ-line cysts stained with thioflavin T, which was reported as a marker of amyloid structures, have shown small fluorescent signals in the NCs, that marked amyloidal structures in the nuage area (Figure 3(d)). A network of long membranous tubules of the endoplasmic reticulum and numerous Golgi complexes that were enclosed by them were also located in the NCs, especially in the cell pole opposite to the RC (Figures 2(b), 3(a)–3(c), 4(g), and 4(h), Supplementary Video 5 online). Analyses of the germ-line cysts in TEM have also shown the nuage material in the cytophore (Figure 3(c) inset).

## 3. Discussion

### 3.1. Mitochondrial Spatial Organization in Syncytial Germ-Line Cysts

Mitochondrial fusion and fission seem to be necessary for a cell's survival and adaptation to changing conditions, and are required for cell growth, division and the distribution of mitochondria during cell differentiation [28, 36]. These opposing processes enable damaged mitochondria to be “rescued” by combining them in a network and compensating for any existing defects and/or eliminating damaged mitochondria (with accumulated mutations) by separating and degrading them via mitophagy (a kind of programmed cell death – autophagy) [19, 72, 73].

Mitochondria can differ markedly in the degree of their connectivity and thus have different morphologies [11]. These differences are the result of the different ratio of fusion to fission events, and based on this proportion, the following states of mitochondria dynamism can be distinguished: (1) fragmented mitochondria in which there is no fusion or fission; (2) micro- and mesofused mitochondria in which fission has the advantage over fusion; (3) dynamic hyperfusion in which fusion has the advantage, but fission also occurs and (4) static hyperfusion in which fission is very rare and mitochondrial dynamism is manifested by a high level of mitochondrial fusion and sometimes only one huge mitochondrion exists [11]. Our observations suggest that in the case of the NC and cytophore that were analyzed, the mitochondria are in a dynamic hyperfusion state (classification and nomenclature were taken from Hoitzing and co-workers, 2015), because in the networks that were analyzed, we did not observe one, large mitochondrion in the NC and cytophore, but rather many extensive, long and branched mitochondria, which were combined with small individual ones. It agrees with the general suggestion that static hyperfusion is a response to cell stresses, such as starvation, while the dynamic hyperfusion state observed here is a way to optimize the production of ATP and select the mitochondrial population [1, 11, 14]. Unlike static hyperfusion, dynamic hyperfusion enables mitochondria to be exchanged between the cyst compartments; because of occurrence of fission, parts of the mitochondrial network can tear themselves away and fuse with another network in a different compartment, or can be destroyed in the process of mitophagy [1, 11, 14].

Because abundant mitochondrial aggregations are characteristic for cells with a high energy demand (even for the special areas of a cell, e.g. the midpiece of the sperm) [19, 66] and also because the process of oogenesis is energy consuming, it is not surprising that extensive mitochondrial networks are also present in the germ-line cysts that were studied. Our observations agree with the well-accepted statement that changes in morphology of the mitochondria affect energy production, and generally that elongated (fused) mitochondria produce more ATP than fragmented populations [19, 27, 74, 75]. In the analyzed NCs, mitochondria formed an extensive interconnected network of tubular organelles surrounding the nucleus and extending on one pole of the cell with smaller mitochondria distributed throughout the cytoplasm. The presence of mitochondria in the close proximity of the nucleus is believed to play a role in regulation of gene expression through production of reactive oxygen species (ROS) [76, 77]. The polarization of mitochondria distribution within NCs may also allow more efficient energy production. Because the majority of mitochondria were located on one pole of the NCs opposite the RCs and thereby on the outside parts of the cysts, it can ensure better oxygen availability necessary for ATP production [78]. An analysis of the distribution of mitochondria within the selected cyst compartments showed that the mitochondrial aggregation (mitochondrial density) in the cytophore was more abundant than in the NC. The mitochondria also passed through the RCs that connected the germ cells with the cytophore. These networks can integrate the entire syncytium and may help in the intensive energy production. A future study of the mitochondrial networks and monitoring their dynamism in the cysts in the other stages of oogenesis could provide more comprehensive data. Linking the distribution of mitochondrial networks with their activity within the syncytial cysts, the polarized cells and common cytophore would also deliver interesting information, as was recently found in clitellate annelid male cysts [27, 79] and female germ and somatic cells in the ovary of the earthworm *Dendrobaena veneta *[80]. In *D. veneta* ovary, not all of the mitochondria were active and the level of mitochondrial activity was different in the specific types of germ cells (oogonia, NCs, oocytes) that were measured; however, it was always lower compared to the mitochondria activity in somatic ovarian cells [80]. Unfortunately, our attempts to monitor the mitochondrial function and activity using available assays (JC-1, MnSOD antibody, MitoTracker Orange CMTMRos, DiOC6(3)) were unsuccessful (see Results section). The reagents used were consistently unable to penetrate into the germ-line cysts. Due to the somatic envelope that tightly covered the cysts, only the somatic cells and the growing oocytes, which are not covered so tightly by somatic envelope, showed labeled mitochondria. However, the morphology of mitochondria in syncytial cysts analyzed in TEM (mitochondria with numerous cristae of low or high electron dense matrix) indicated their high activity. So far we can only speculate the large aggregations of mitochondria in the NCs and especially in the cytophore, the significant degree of mitochondrial connections in the form of extensive networks may also play a role in energy production and its better application for more effectively powering/supporting the growing oocyte.

The dynamism of the morphology of mitochondria and thus their behavior during the formation of the future egg cells (i.e. during oogenesis) affects the transmission of these organelles to the offspring (and hence the inheritance of mitochondrial DNA). Therefore, maintaining mitochondria in a good condition by eliminating damaged ones and/or their selection mechanisms during oogenesis is crucial to the health of the offspring [81, 82]. This is especially important because, as is well known, all mitochondria in future organisms are inherited from only the oocyte [83] with only a few exceptions that occur in some bivalves [84, 85].

It should be remembered that although NCs are specific types of germ cells that do not have the potential to develop into functional gametes, they play important roles in oocytes formation, providing them with a lot of structures – organelles and macromolecules such as ribonucleoproteins (RNPs) [43, 86]. Because these components stored in oocyte are crucial for the proper embryo development, they should be protected from any hazardous conditions. It has been shown that the formation of mitochondrial networks is connected with lower production of ROS, which are known to be a major factor that can damage the cell components. One of the proposed functions of a mitochondrial network is to provide antioxidative protection, which prevents cell death and mitophagy [28, 66–68]. The unresolved question is whether the mitochondria in the NCs are the source of mitochondria for future embryo, whether they are packed into the cytophore and degenerate as oogenesis comes to the end. Numerous single mitochondria were observed among the extensive mitochondrial networks of the NC and cytophore, and single autophagy vesicles (autophagosomes) containing remnants of mitochondria and other cell organelles were noted in the NCs and younger germ cells. It suggests that germ cells may select and eliminate the deleterious mitochondria by undertaking the process of their elimination (mitophagy). The mitochondrial networks may thus be engaged in splitting the mitochondria and eliminating them from the networks. There were no signs of mitophagy in the cytophore cytoplasm in the analyzed cysts. On the other hand, it is puzzling what is the cause of the large aggregation of mitochondrial networks just in the cytophore. In the male cysts equipped with a cytophore, the cytophore serve as a place for cytoplasm remnants during the process of sperm formation and their release from the cysts [87]. In the cytophore in cysts containing late spermatids, large aggregations of mitochondria were formed and finally they are removed together with the cytophore and the cyst remnants [71]. In female germ-line cysts however, the cytophore acts as an intermediary structure between NCs and the growing oocyte, and it is the route by which the cell organelles and macromolecules are transferred [52, 88]. Considering the fact that the oocyte gathers a large amount of mitochondria when it grows, the cytophore may be a place where the selected mitochondria are stored and finally pass into the ooplasm. Additionally, as was shown in the previous study, in late oogenesis in *E. albidus* a large vitellogenic oocyte surrounds the rest of the cyst and it was suggested that the oocyte can engulf it at the end [52].

### 3.2. Nuage Material as an Equivalent of the Balbiani Body?

In the oocytes of many animals, the mitochondria are organized into a transient structure called a Balbiani body (mitochondrial cloud; Bb). In addition to mitochondria, the Bb is composed of other cell structures such as the endoplasmic reticulum, Golgi complexes and nuage accumulations [67, 89, 90]. Nuage is an omnipresent component of germ-line cells [67, 91]. In brief, it derives from the nucleus and thus it constitutes a source of RNPs and is involved in correct localization of various mRNAs in the germ plasm (i.e. the determinants of the germline) in the oocytes [32, 51, 53, 54, 92]. The relationships between the Bb constituents has recently been shown in the oocytes of the insect *Thermobia domestica*, and in the context of mitochondrial dynamism, the fusion of mitochondria in a network and the fission of single mitochondria from these aggregations were demonstrated. This mechanism is believed to select and eliminate dysfunctional mitochondria from the future egg cells [31]. Although the Bb has not been described in Annelida, in many of the annelid species that have been studied to date, a granulo-fibrillar electron-dense nuage material was found in both the oocytes and NC [72, 73, 93]. Our observations of a dense mitochondrial network that was closely related not only to the nuage material but also to endoplasmic reticulum and Golgi complexes suggest that such aggregations may be a less morphologically prominent structural equivalent of the Bb (occurring in the entire germ-line cyst, not only in oocyte). The presence of nuage material within the cytophore cytoplasm indicates that the NC may be involved in producing and transporting the germ plasm determinants to the oocyte. Although the composition of nuage material in annelids is not known so far, it is worth noting that it contains amyloidal structures, which was confirmed by thioflavin T staining. Despite the lack of an appropriate, structurally well separated Bb in annelids, the dispersed nuage material and dense mitochondrial networks that stretch through the cyst components may compensate for it. Accumulations of nuage material with cell organelles can also be expected in annelid oocytes and their analysis should be the next step in future studies. It is well accepted that induction of germ-line fate may be of two origins: zygotic induction or maternal cytoplasmic inheritance [92, 94, 95]. The first situation takes place during embryogenesis and is initialized by cell-to-cell signaling; it is widespread among animal taxa and may represent the ancestral mechanism. The second way of germ line origin is based on germ-line determinants, as mRNAs and proteins, which are deposited during oogenesis in the specific region (germ plasm) of the oocyte. The formation of the germ-line by inheritance of maternal information was found in such model system as *C. elegans*, *D. melanogaster *and *X. laevis*, and it is believed to be a derived condition [92, 94, 95]. The presence of the nuage material in the NCs and the cytophore represents the maternal determination of germ-line fate. It indicates that the germ-line determinants, although initially spread in all of the cyst's cells, will be finally transferred via the cytophore into the future gamete. A similar situation takes place e.g. in *D. melanogaster*, in which germ-line determinants are produced in NCs, transported via ring canals to the oocyte and after that they become localized in the posterior pole of the oocyte, forming the pole plasm. Moreover, germ plasm is believed to host healthy mitochondria during development to pass on to future generations [96]. In animals with zygotic induction of the germ-line such as vertebrates, the Balbiani body is also formed, and its suggested role is protection of the quality of mitochondria and other organelles during long lasting oogenesis [68, 96]. Staining of *E. albidus* germ cells with thioflavin T dye, which stains the beta sheet-rich structures of amyloid, [96, 97] revealed that nuage contains amyloid-like structures. Similar structures were recently observed and analyzed in *Xenopus* oocytes. In this model species, Xvelo, a highly enriched protein in Balbiani bodies, is rich in amyloid-like assemblies [96]. This amyloid-rich protein also has a prion-like domain which, as was shown experimentally, is suggested to structurally organize Balbiani bodies. This domain can form a stable matrix, in which mitochondria and other organelles are embedded, and may be engaged in binding and concentration of organelles and RNA [96]. In *E. albidus*, the close contact of nuage material with mitochondria may thus suggest its role in protection of the quality of mitochondria and may cluster them together in mitochondrial-nuage aggregations, which may pass together via ring canals and move further. It could be suggested that amyloid-like proteins may form an evolutionary conserved mechanism involved in some way in specification of the germ-line, as evidenced by e.g. *oskar* RNA in *D. melanogaster*, which is one of the major proteins involved in pole plasm organization [89, 91, 98], and shows similarity with *Xenopus* Xvelo [96], and also by amyloid-like aggregation of RNA-binding proteins in yeast, which are important in regulation of gametogenesis and sexual reproduction [99].

To sum up, the observations presented here are the first step in studying the process of mitochondrial dynamism within syncytial germ-line cysts with a common cytoplasm. The serial block-face SEM method that was used enabled us to analyze the distribution of mitochondria and their correlative organelles and structures using high resolution three-dimensional reconstructions for the first time. Using SBEM and other microscopic methods it was demonstrated that:

- The NCs are polarized – dense accumulations of mitochondria are located near the nuclei in the pole of the cell opposite to the RCs.
- In the cytophore there is 25% more abundant accumulation of the mitochondria, compared to the nurse cell and the mitochondria are distributed evenly in the cytoplasm.
- The mitochondria are connected into a few large and numerous less connected organelles, between which small individual mitochondria also occurs; the level of the mitochondria connectedness versus individual ones indicated that they are in dynamic hyperfusion state.
- The mitochondria can pass through the RCs; thus they can be exchanged between particular cyst compartments.
- The dispersed nuage material seems to be the structural equivalent of the Bb.

## 4. Materials and Methods

### 4.1. Animal Material

Specimens of the white worm *Enchytraeus albidus* (Henle, 1837) were obtained from commercial sources. They were bred under laboratory conditions in plastic boxes filled with potting soil. They were fed once a week with bread and vegetables soaked in water. Only mature specimens with a clearly visible clitellum were used for the analyses.

### 4.2. Preparing the Material for Analysis Using Differential Interference Contrast and Fluorescent Microscopy

Specimens of *E. albidus* were fixed in 4% formaldehyde (freshly prepared from paraformaldehyde) in PBS (phosphate buffered saline, NaCl, 137 mM; KCl, 2.7 mM; Na_2_HPO_4_ 8 mM; KH_2_PO_4_, 1.5 mM, pH 7.4) for 30–40 min at room temperature and washed in PBS. The ovaries containing germ-line cysts were dissected, mounted on microscopic slides and analyzed under an Olympus BX60 microscope equipped with Nomarski differential interference contrast, or double stained with rhodamine-conjugated phalloidin (2 *μ*g/ml; Sigma) and DAPI (1 *μ*g/ml) for 40 min in darkness, washed in PBS and analyzed under the same microscope equipped with the appropriate filters.

For detection of amyloid-like aggregations in germ cells, the body parts with gonads were fixed as above, dehydrated for 15 min in a series of 30%, 50%, 70%, 96%, and 100% ethanol solutions, saturated in ethanol/Steedman wax solutions: in 3 : 1, 1 : 1, 1 : 3 and 100% ethanol for 24 h each. Then the material was embedded in Steedman wax, left for polymerization and cut into 7 *μ*m thick sections on a Zeiss HYRAX M40 microtome. Before staining, the sections were mounted onto microscope slides and dewaxed using a reverse series of ethanol: 100% for 2 × 15 min and 90, 70 and 50%, double-distilled water (ddH_2_O) for 15 min each. The de-waxed sections were then washed with 1% Triton X-100 in PBS and then in pure PBS and afterwards incubated for 1 h in 1% BSA (bovine serum albumin) in PBS. The material was stained in 1% aqueous thioflavin T for 20 min at room temperature in darkness. The stained tissue sections were analyzed under an Olympus BX60 microscope equipped with appropriate filters.

### 4.3. Preparing the Material for Analysis Using Light Microscopy, Transmission Electron Microscopy and SBEM

The dissected body segments with gonads were fixed in 2.5% glutaraldehyde in a 0.1 M phosphate buffer (pH 7.4) at room temperature for 1 h. After washing in the same buffer, the samples were post-fixed with 3% potassium ferrocyanide in a 0.3 M cacodylate buffer mixed with an equal volume of a 4% aqueous solution of osmium tetroxide for 1 h. The tissue was then washed three times for 5 min in ddH_2_O and incubated in a 1% solution of thiocarbohydrazide (Ted Pella) for 20 min at 60°C. After that, the samples were washed three times for 5 min in ddH_2_O and placed in 2% aqueous osmium tetroxide for 30 min, then the tissue was washed again three times for 5 min in ddH_2_O and incubated overnight in 1% aqueous uranyl acetate at 4°C. The samples were then rinsed three times for 5 min in ddH_2_O and put into freshly prepared Walton's lead aspartate for 30 min at 60°C, washed five times for 3 min in ddH_2_O and dehydrated for 10 min in a series of 30, 50, 70, and 96% ethanol solutions, then placed in anhydrous 100% ethanol three times for 20 min, a 1 : 1 solution of acetone and ethanol for 15 min and twice for 15 min in 100% acetone. After dehydration, the samples were placed in a mixture of 50% Epoxy Embedding Medium (Sigma-Aldrich, St. Louis, MO, USA) in acetone for 3 h, then left overnight for the acetone to evaporate. The prepared material was embedded between two layers of Aclar (EMS) and left to polymerize.

To analyze the material using light microscopy, semithin sections (0.8 *µ*m thick) were stained with methylene blue and analyzed using an Olympus BX60 microscope equipped with an XC50 digital camera (Olympus, Tokyo, Japan) and cellSens Standard software (Olympus, Tokyo, Japan). To analyze the material using transmission electron microscopy, ultrathin sections (80 nm) were cut on a Leica Ultracut UCT ultramicrotome (Leica Microsystems, Wetzlar, Germany) and examined under a Hitachi H500 transmission electron microscope (Hitachi, Tokyo, Japan) at 75 kV.

For the 3D reconstructions, after resin hardening, squares, cut out with razor-blades, were attached to aluminum pins (metal rivets, Oxford Instruments) with a very small amount of cyanoacrylate glue and then mounted to the ultramicrotome (Ultracut UCT ultramicrotome, Leica Microsystems, Wetzlar, Germany) and the block of the sample was trimmed. Next, samples were grounded with silver paint (Ted Pella, 16062-15) to the pin and dried for 24 h. Stacks of images from serial 150 nm ultrathin sections were collected using a Sigma VP (Zeiss) scanning electron microscope equipped with ultramicrotome chamber 3View (Gatan) and Digital Micrograph software (Gatan) and back scattered electron detector. Scanning parameters: variable pressure 18 Pa, EHT 4 kV, aperture 15 *μ*m, dwell time 7 *μ*s, pixel size 15 nm.

### 4.4. Three-Dimensional Reconstructions

Three-dimensional reconstructions of the selected cyst components were based on a series of ultrathin sections. The Microscopy Image Browser (MIB), which is a handy tool for image management [100], was used to prepare the three-dimensional models. Interesting cell compartments were segmented manually in MIB using a brush and threshold tool. The models that were obtained were visualized in a trial version of Amira (Thermo Scientific, Waltham, MA. USA).

### 4.5. Measurement of the Mitochondria

Based on the three-dimensional reconstructions of a series of ultrathin sections the mitochondria and mitochondria aggregates were segmented using the surface object detection function built in Imaris (custom software developed by Bitplane Scientific Software, Zurich, Switzerland). The same methodology was used separately in nurse cell and cytophore. The cytophore, nurse cell, and ring canal were segmented using surface modeling, which is available in Imaris with a manual outline. In each detected mitochondria aggregates object number of mitochondria was calculated using an estimated fixed mitochondrion size. The mitochondrion size was determined by precisely manually contoured several single mitochondrion shapes on a series of ultrathin sections. 3D objects were constructed using surface object detection and for each object the volume estimate was calculated. The average volume value of 25 randomly selected objects was used to determine the final mitochondrion size. Using the calculated volume of the mitochondria aggregates and estimation of the single mitochondrion, the mitochondria number in each aggregate was calculated. Independent calculations of the total volume of the mitochondria elements in relation to the volume of the cytophore and nurse cell, respectively, allowed to determine the relative mitochondria concentration ratio in cytophore with regards to nurse cell.

The 3D position coordinates of all of the mitochondria that were detected inside the cytophore and nurse cell were measured. As a result, we calculated the Euclidian distances between each mitochondrion and the center of the ring canal in the cytophore and nurse cell. The distances that were calculated were normalized by the maximal length of the cytophore or nurse cell based on the localization of the mitochondria. Statistical analysis was performed using the R environment (ver. 3.4.2).

## Figures and Tables

**Table 1:** Measurement of the volume of mitochondria and their comparison in the nurse cell and cytophore that were analyzed.

	Nurse cell	Cytophore
Cytoplasm volume	10,477.96 *µ*m (12,241.4 cell volume−1,763.44 nucleus volume)	1,916.66 *µ*m

Volume of mitochondria	964 *µ*m	221 *µ*m

Percentage of volume of mitochondria per cytoplasm	9.2%	11.5%

~ Ratio of the mitochondria volume	1 : 1.25	

## Data Availability

The data used to support the findings of this study are available from the corresponding author upon request.

## Supplementary Materials

Supplementary 1 Supplementary Video 1: video of sequential ultrastructural micrographs of analyzed germ-line cyst, obtained by SBEM technique, which was the basis for the 3D visualizations.

Supplementary 2 Supplementary Video 2: visualization of the reconstructed nurse cell and cytophore with the mitochondria visible in the cell (*dark blue*) and within the cytophore (*light green*). Mitochondria (*yellow*) passing through the ring canal (*light blue*), nurse cell nucleus (*blue*).

Supplementary 3 Supplementary Video 3: visualization of the 16 ring canals: 15 from nurse cells (*light blue*) and one from oocyte (*red*) in the germ-line cyst with the mitochondria (*yellow*) passing through them. Cytophore mitochondria (*light green*).

Supplementary 4 Supplementary Video 4: zoom of the reconstructed ring canal (*red*) that connects the oocyte to the cytophore with the mitochondria passing through it (*yellow*).

Supplementary 5 Supplementary Video 5: visualization of the region in nurse cell with nuage material. Nurse cell nucleus (*blue*), nuage material (*light green*), mitochondria (*dark red*), Golgi complexes (*purple*), endoplasmic reticulum (*light blue*).

Supplementary 6 Supplementary Figure 1: (A)–(C) Live-cell imaging of germ-line cysts stained with (A) DiOC to mark mitochondria (*green*), bar = 50 *µ*m (B) MitoTracker Orange CMTMRos to stain mitochondria (*red*) and Hoechst 33342 to counterstain the cell nuclei (*blue*), bar = 50 *µ*m (C) JC-1 to visualize the active (*green*) and nonactive (*red*) mitochondria and Hoechst 33342 to counterstain the cell nuclei (*blue*), bar = 40 *µ*m (D) Immunolabeling with the MnSOD antibody to detect mitochondrial superoxide dismutase (*green*), bar = 50 *µ*m. NC – nurse cells, O – oocyte.
