# Supplementary figures and images for: Morphology of Mitochondria in Syncytial Annelid Female Germ-Line Cyst Visualized by Serial Block-Face SEM
Source: Int J Cell Biol. 2020 Jan 7;2020:7483467. doi: 10.1155/2020/7483467 (PMC7199535; doi:10.1155/2020/7483467)

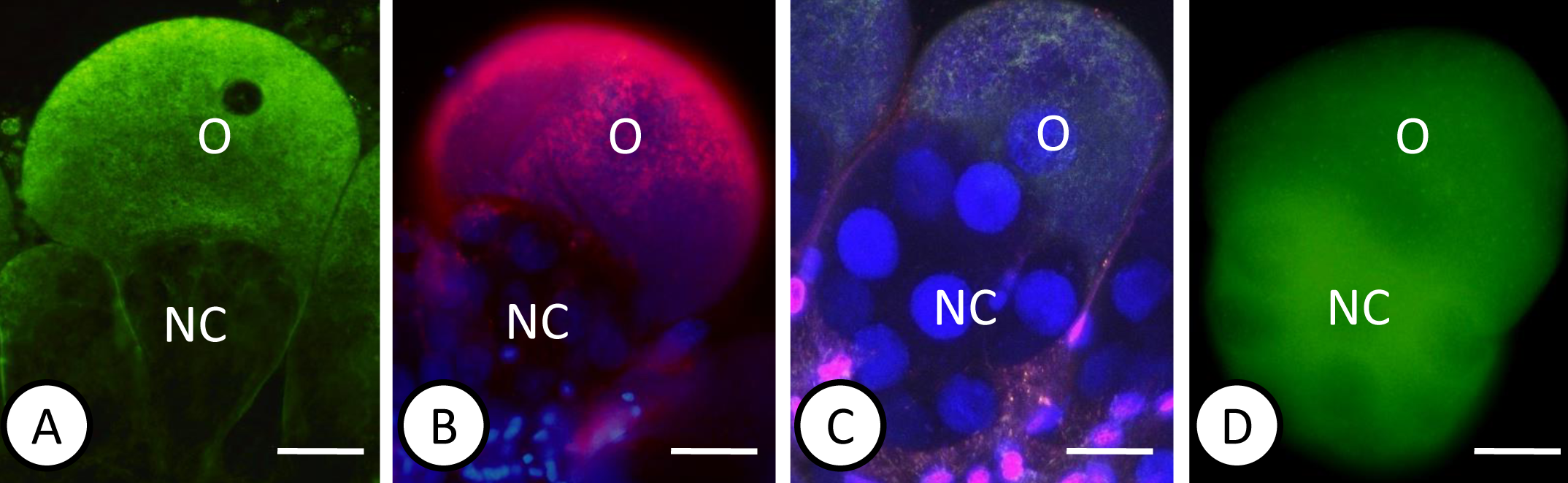

Supplement: Supplementary 6 — Supplementary Figure 1: (A)–(C) Live-cell imaging of germ-line cysts stained with (A) DiOC to mark mitochondria (green), bar = 50 µm (B) MitoTracker Orange CMTMRos to stain mitochondria (red) and Hoechst 33342 to counterstain the cell nuclei (blue), bar = 50 µm (C) JC-1 to visualize the active (green) and nonactive (red) mitochondria and Hoechst 33342 to counterstain the cell nuclei (blue), bar = 40 µm (D) Immunolabeling with the MnSOD antibody to detect mitochondrial superoxide dismutase (green), bar = 50 µm. NC – nurse cells, O – oocyte. [file 7483467.f6.tif]
